# A Catalytic Sensor for Measurement of Radical Density in CO_2_ Plasmas

**DOI:** 10.3390/s121216168

**Published:** 2012-11-22

**Authors:** Alenka Vesel, Rok Zaplotnik, Jonathan Iacono, Marianne Balat-Pichelin, Miran Mozetic

**Affiliations:** 1 Jozef Stefan Institute, Jamova 39, 1000 Ljubljana, Slovenia; E-Mails: alenka.vesel@ijs.si (A.V.); rok.zaplotnik@ijs.si (R.Z.); 2 PROMES-CNRS Laboratory, 7 rue du Four Solaire, 66120 Font-Romeu Odeillo, France; E-Mails: jonathan.iacono@promes.cnrs.fr (J.I.); marianne.balat@promes.cnrs.fr (M.B.P.)

**Keywords:** catalytic probe, plasma probes, atom measurement, dissociation fraction measurement, carbon dioxide plasma

## Abstract

A catalytic sensor for the measurement of radical density in weakly ionized CO_2_ plasmas, created in a low-pressure electrodeless discharge, is presented. The CO_2_ plasma was created in a 4 cm wide borosilicate glass tube inside a copper coil connected to a radio frequency generator operating at 27.12 MHz with a nominal power of 250 W. The dissociation fraction of the CO_2_ molecules was measured in the early afterglow at pressures ranging from 10 Pa to 100 Pa, and at distances of up to 35 cm along the gas stream from the glowing plasma. The radical density peaked (2 × 10^20^ m^−3^) at 80 Pa. The density quickly decreased with increasing distance from the glowing plasma despite a rather large drift velocity. The dissociation fraction showed similar behavior, except that the maximum was obtained at somewhat lower pressure. The results were explained by rather intense surface recombination of radicals.

## 1. Introduction

Oxidative plasmas are a popular medium for modification of the surface properties of solid materials. Chemically reactive particles created in the oxidative plasma react with solid materials at much lower temperature than the parent molecules, and this allows for surface modification of the materials at relatively low temperature. This treatment often leads to unique surface properties for the treated materials. Hydrophobic materials may become hydrophilic [1,2] and sometimes superhydrophilic [3]. When the surface morphology is modified [2–4] metals form a thin oxide film [5], sometimes in the form of nanowires and other thermodynamically unfavorable forms [6,7], and composite materials are etched preferentially [8,9]. Oxidative plasmas are usually created in oxygen (O_2_) or a mixture of O_2_ with a noble gas, such as argon or helium. These plasmas are often very reactive and may destroy delicate samples through thermal degradation caused by exothermic reactions that take place on the material surface.

Plasmas can be created with different gaseous discharges at different powers. Powerful discharges are suitable for production of large quantities of metal oxide nanoparticles [7,10]. However, they are too aggressive for treatment of polymer materials, let alone delicate biomedical samples [11]. In such cases, it is best to use plasma at a very low neutral gas kinetic temperature, relatively low ionization fraction, and moderately high dissociation fraction. Electrodeless high frequency discharges seem to be particularly useful for the generation of plasmas with these characteristics. A small problem arising from application of plasmas created in discharges at low power is the ignition phenomenon. It has been known for a century that the breakdown power of many electrodeless discharges is much larger than the sustaining power. There are several methods for overcoming this problem, including application of powerful pulses for ignition of the discharge. Another possible solution is application of an early afterglow instead of the plasma itself. The density of many plasma particles is much lower in early afterglow than in glowing plasma, while some other particles appear to be relatively stable and their density in the early afterglow is not much lower than that seen in the plasma itself. This is especially true for neutral O atoms, where the decay length in high frequency discharges may be very long [12]. A less aggressive alternative to O_2_ is application of plasma created in a gas that contains O atoms, such as water vapor or carbon dioxide (CO_2_). Although it was demonstrated that CO_2_ plasmas have certain advantages in polymer surface functionalization applications [13–15], very little work has been published on the decay of the dissociation fraction in an early afterglow of plasmas created in CO_2_[16]. In the present paper, we address this phenomenon. For this purpose a thermocouple nickel catalytic probe was used. This probe is usually used for measuring the densities of neutral atoms produced in an electromagnetic discharge of diatomic gas molecules, such as oxygen and hydrogen. However with some modifications of the heterogeneous recombination model on a probe, radical densities in an early afterglow of a plasma created in a CO_2_ electrodeless radiofrequency discharge can also be determined.

## 2. Experimental Section

**Figure 1.**
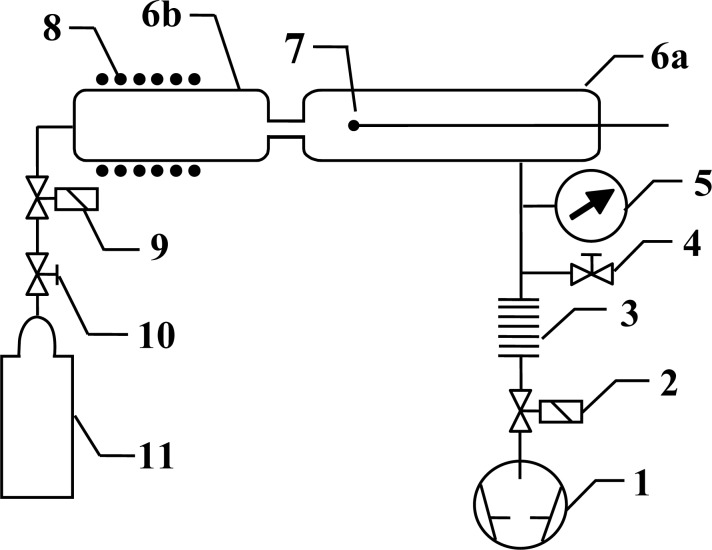
Experimental setup: 1. rotary pump; 2. gate valve; 3. Hopkins trap; 4. air inlet valve; 5. vacuum gauge; 6a. discharge chamber; 6b. post-discharge chamber; 7. catalytic probe; 8. RF coil; 9. leak valve; 10. high-pressure valve; 11. carbon dioxide.

**Figure 2.**
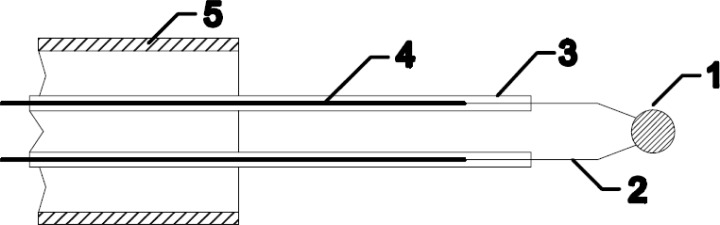
Thermocouple catalytic probe: 1. nickel disk; 2. thermocouple wires; 3. thin glass tube; 4. kovar wires; 5. glass tube.

Experiments were performed using the discharge system shown in Figure 1. The main component of this system is a discharge tube (I.D. 3.6 cm), which is connected to an afterglow tube (I.D. 3.6 cm) by a narrow glass tube (I.D. 7 mm, length 6 cm) as shown in Figure 2. Carbon dioxide leaks into the discharge system through a manually adjustable high vacuum leak valve. The gas passes into the discharge region where it is transformed into plasma, and then continues into the afterglow chamber through a narrow borosilicate glass tube. The narrow tube effectively separates the glowing plasma from the early afterglow. The intensity of the emitted light is uniform in the discharge tube and diminishes as the gas enters the narrow tube. Visibly glowing plasma is present in the first 3–5 cm of the narrow tube, with the exact distance depending on the pressure, and converts to early afterglow thereafter. The absence of plasma glow results from the loss of electrons because of surface neutralization along the narrow tube.

The gas in the afterglow continues its way toward the pump passing through the vacuum gauge (Baratron® Absolute, pressure range 1–1,000 Pa, MKS Instruments, Andover, MA, USA) and flowing through a recombinator until it finally reaches the pump. The CO_2_ pressure was varied between 20 Pa and 120 Pa. The recombinator contains a rough copper mesh that effectively recombines any radicals into stable molecules, but this has practically no influence on the gas flow in the sense of lowering the effective pumping speed. Without the recombinator, radicals might react with the oil in the rotary pump. The nominal pumping speed of the two-stage rotary pump was 16 m^3^ h^−1^.

**Figure 3.**
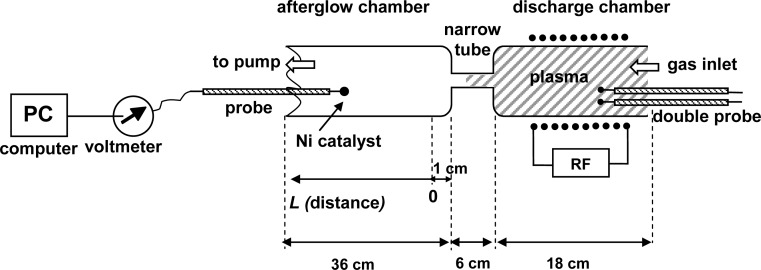
Details of the plasma and afterglow reactors.

A standard thermocouple nickel catalytic probe [12] (Figure 2) was mounted into the afterglow chamber as shown in Figure 3. Detailed operation of catalytic probe and calculations for determination of neutral oxygen atoms in oxygen plasma afterglow is described elsewhere [17]. The temperature of the catalytic probe, which is needed for calculation of the density of radicals, was measured by a voltmeter connected to a computer. The catalytic probe was movable so that it was possible to perform measurements with the probe tip at different positions in the afterglow chamber. Before the experiments with the catalytic probe, a simple double electrical probe was mounted into the center of the discharge tube to estimate the density of positive ions in the glowing plasma. The saturated ion current depended on the pressure and slowly decreased with increasing pressure. The order of magnitude for the density of charged particles was 10^16^ m^−3^.

## 3. Results and Discussion

**Figure 4.**
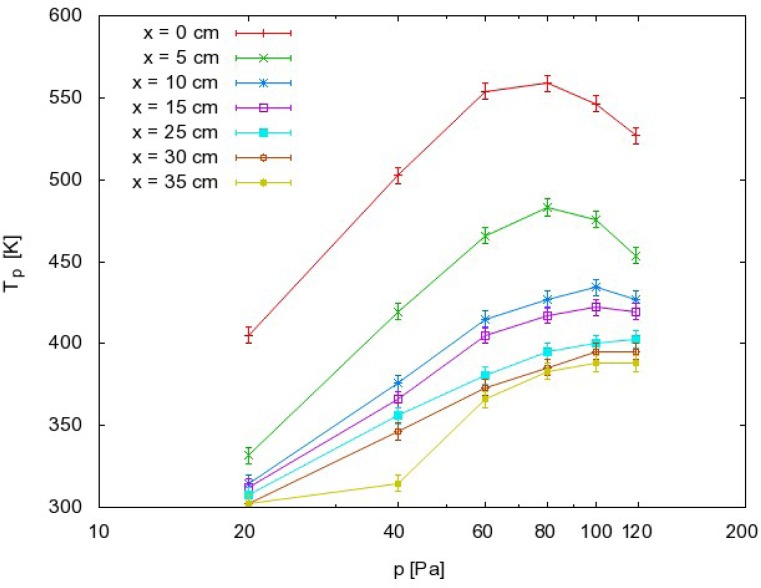
Maximum temperature of a nickel catalyst versus pressure with the catalytic probe at different positions in the afterglow chamber. Absolute error is about ≈ 5 K due to instrument (voltmeter) error.

Systematic measurements were performed by adjusting the pressure in the afterglow chamber to an appropriate value, and then moving the probe along the afterglow chamber. The zero position was approximately 1 cm away from the exit of the narrow tube (see Figure 3). Measurements were performed at discrete positions separated by 5 cm. The maximum temperature of the probe under various experimental conditions is presented in Figure 4. The probe temperature measurements are both reliable and reproducible, while only the thermocouple and voltmeter are used. These measurements are also very accurate, with a precision of about ≈ 5 K. The density of the radicals was then calculated using the standard procedure, which is based on heating of the probe because of recombination of plasma radicals on its surface. One must also consider the heating contribution of ions and plasma radiation. However in our case there were no ions in the afterglow chamber, as they all were neutralized in the narrow tube. The role of plasma irradiation on the probe was measured with an optical emission spectrometer (OES) with sensitivity of 14,000 counts μW^−1^ per ms integration time. We measured OES spectra near the probe position and we estimated the radiation power to be about 10 W. This means that the nickel disk with mass of 2.6 × 10^−6^ kg and 444 J·kg^−1^·K^−1^ thermal heat capacity would heat up for 1 K in approximately 120 s. Even though this is an overestimation, while the solid angle of the probe is at least ten times lower than solid angle of spectrometer, the contribution from plasma irradiation can be neglected while the probe heats up, usually to about 100 K (Figure 4) in 20 s or less. Therefore the probe is heated mostly through the heterogeneous reactions of plasma radicals.

Here it is worth mentioning that we had to activate the probe. The nickel probe tip was oxidized and because of that, the first few measurements in an afterglow of the CO_2_ plasma were not reproducible. Several measurements had to be done to achieve reproducibility. This could be explained with the adsorption of CO molecules on the surface of the nickel disk. Thus the heterogeneous reactions on the surface of the probe tip are [18]:

(1) $$\text{O}+\text{O}\to {\text{O}}_{2}$$

(2) $$\text{CO}+\text{O}\to {\text{CO}}_{2}$$

Reactions (1) and (2) include both the Langmuir-Hinshelwood (O_ads_ + O_ads_ → O_2_, CO_ads_ + O_ads_ → CO_2_) and Eley-Rideal mechanisms (O + O_ads_ → O_2_, CO + O_ads_ → CO_2_, CO_ads_ + O → CO_2_), while for determination of radicals only power released by Reaction (1) and power released by Reaction (2) are needed. The recombination mechanism is not important for our calculations. Recombination probabilities γ used for determination of radicals are therefore averaged probabilities for both mechanisms.

Because the probe is exposed to fluxes of both O atoms and carbon monoxide (CO) molecules, the released power on the surface of the probe can be simply and roughly described by the following Equation:

(3) $$P=½\cdot \gamma_{{\text{O}}_{2}}\cdot W_{{\text{D}}_{{\text{O}}_{2}}}\cdot (j_{\text{O}}-\gamma_{{\text{CO}}_{2}}\cdot j_{\text{CO}})\cdot A+½\cdot \gamma_{{\text{CO}}_{2}}\cdot W_{{\text{D}}_{{\text{CO}}_{2}}}\cdot 2j_{\text{CO}}\cdot A$$

where γ_O_2__ and γ_CO_2__ are the recombination probabilities for Reactions (1) and (2), respectively; *W*_D_O_2___ and *W*_D_CO_2___ are dissociation energies of O_2_ and CO_2_ molecules (*i.e*., *W*_D_O_2___ = 5.2 eV and *W*_D_CO_2___ = 5.5 eV [19]); *j*_O_ and *j*_CO_ are the fluxes of O atoms and CO molecules, respectively, to the probe surface with area *A* (1.4 × 10^−5^ m^2^). The first part of Equation (3) is the contribution from recombination into oxygen molecules, where the flux part is the O atoms flux minus the O atoms flux used for CO_2_ recombination. The second part of Equation (3) is the contribution from recombination into carbon dioxide molecules, where the flux part is two times CO flux whereas the flux of O atoms used for CO_2_ recombination is the same as the flux of CO molecules. The factor ½ arises from the fact that two particles, two atoms (O) or one molecule (CO) and one atom (O), are needed to recombine into one molecule O_2_ or CO_2_, respectively.

The fluxes *j*_O_ and *j*_CO_ are defined as follows:

(4) jO=14⋅nO⋅vO

(5) jCO=14⋅nCO⋅vCO

where *n*_O_ and *n*_CO_ are the densities of O atoms and CO molecules. The average thermal velocities of the O atoms and CO molecules, *v*_O_ = 630 m/s and *v*_CO_ = 476 m/s, are calculated using $v=\sqrt{8k_{\text{B}}T/(\pi \cdot m)}$. Because the CO_2_ molecule dissociates to equal amounts of CO and O, and because the probability for heterogeneous recombination of O atoms on glass surfaces is very low, around 10^−4^[20], the densities of CO and O in a plasma afterglow can be considered as equal (*i.e*., *n*_O_ = *n*_CO_). Combination of Equations (3), (4) and (5) provides the following formula:

(6) $$P=\frac{A\cdot n_{\text{O}}}{8}\left(\gamma_{{\text{O}}_{2}}\cdot W_{{\text{D}}_{{\text{O}}_{2}}}\cdot (v_{\text{O}}-\gamma_{{\text{CO}}_{2}}\cdot v_{\text{CO}})+\gamma_{{\text{CO}}_{2}}\cdot W_{{\text{D}}_{{\text{CO}}_{2}}}\cdot 2v_{\text{CO}}\right)$$

The probe heating can be calculated as follows:

(7) $$P={\mathrm{mc}}_{p}\frac{\text{d}T}{\text{d}t}$$

where *m* is the mass of the probe, *c_p_* is its thermal heat capacity, and d*T*/d*t* is the time derivate of the probe temperature just after the heating source has been turned on. The density of O atoms can be thus calculated from a combination of Equations (6) and (7) as follows:

(8) nO=8⋅m⋅cp⋅(dTdt)A⋅(γO2⋅WDO2⋅(vO−γCO2⋅vCO)+γCO2⋅WDCO2⋅2vCO)

Recombination probability γ_O_2__ is well known and for nickel surfaces it is 0.27, while the recombination probability for γ_CO_2__ is not known. Most studies take into account the average heterogeneous surface recombination probability [19] or assume γ_O_2__ = γ_CO_2__ [18]. We made the same assumption in the present paper.

**Figure 5.**
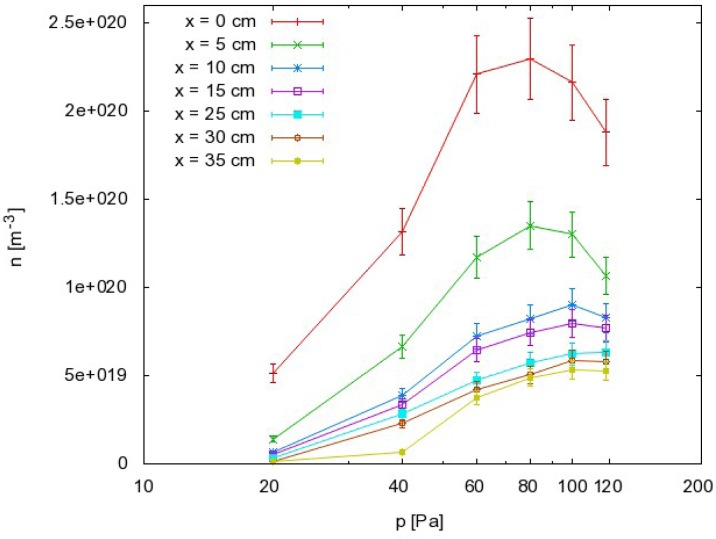
Oxygen atom density versus pressure at different positions in the afterglow chamber. Relative error is around ≈ 10% due to the temperature measurement error and probe physical characteristics measurement error (surface area, mass).

**Figure 6.**
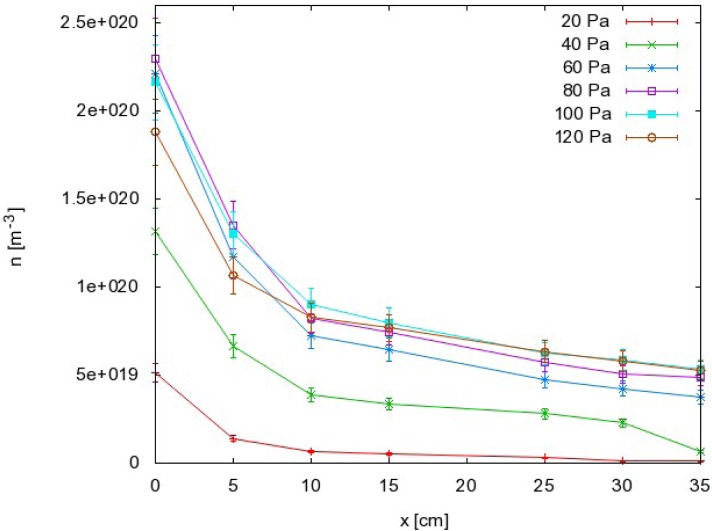
Oxygen atom density along the afterglow chamber at different pressures. Relative error is around ≈ 10%.

The results as calculated from Equation (8) are summarized in Figure 5, where the density of O atoms is plotted against the pressure at different distances along the narrow tube. The corresponding graph of *n*_O_ against the distance is presented in Figure 6.

**Figure 7.**
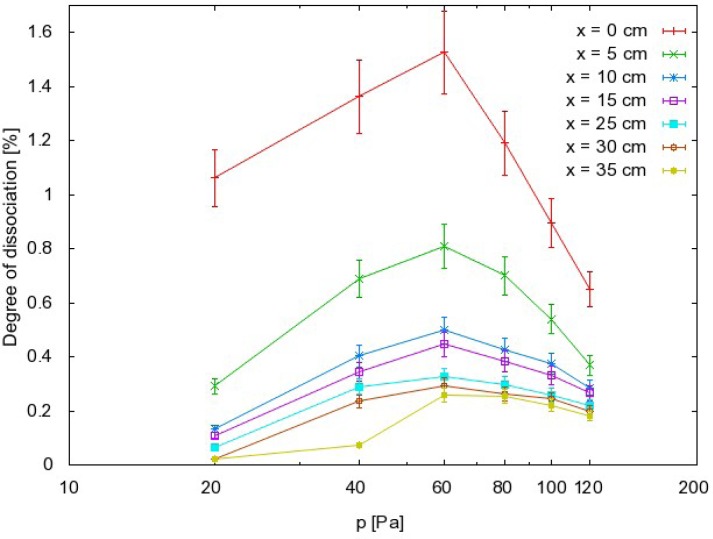
Dissociation fraction of CO_2_ molecules in the afterglow chamber versus pressure. Relative error is the same as in density results (≈ 10%), while error in $p_{{\text{CO}}_{2}}^{0}$ measurement is negligible.

Finally, the dissociation fraction of CO_2_ molecules can be calculated from the following equation:

(9) $$\eta =\frac{n_{\text{O}}}{n_{{\text{CO}}_{2}}^{0}}=\frac{n_{O}kT}{p_{{\text{CO}}_{2}}^{0}}$$

where *k* is the Boltzmann constant, *T* is the neutral gas kinetic temperature, and $p_{{\text{CO}}_{2}}^{0}$ is the pressure of CO_2_ before the discharge is turned on. The results are summarized in Figure 7.

The results reveal some interesting behaviors that can be summarized as follows:

- the order of magnitude for the dissociation fraction is about 1%;
- the dissociation fraction exhibits a rather broad maximum at 60 Pa;
- the O atom density also exhibits a maximum but it is slightly shifted to higher pressure compared to the dissociation fraction;
- there is a sharp drop in both the dissociation fraction and atom density in the first few centimeters along the narrow tube exhaust.

The dissociation fraction of CO_2_ molecules in the early afterglow is rather low, especially when compared with experiments performed in pure O_2_ or hydrogen (H_2_) under very similar discharge conditions [21,22]. The value obtained in the present study was at least an order of magnitude lower than those obtained in the earlier studies. For example, in pure O_2_ the neutral O atom density exceeded 5 × 10^21^ m^−3^. The differences between our results and those in the earlier studies can be explained either by different production or loss rates of radicals. Because the plasma density is practically the same as those in experiments with O_2_, the only explanation for the reduced O atom production is a lower dissociation rate. The dissociation energies of O_2_, H_2_, and CO_2_ molecules are 5.2, 4.5 and 5.5 eV, respectively. Because the dissociation energy of carbon molecules is not much larger, it cannot be the reason for poor production. CO_2_ dissociation can occur by electron impact dissociation, dissociative recombination of positive ions, and dissociation stimulated by vibrational excitation of molecules [16] as follows:

(10) $${\text{CO}}_{2}+{\text{e}}^{-}\to \text{CO}+\text{O}+{\text{e}}^{-}$$

(11) $${\text{CO}}_{2}^{+}+{\text{e}}^{-}\to \text{CO}+\text{O}$$

(12) $${\text{CO}}_{2}^{\text{v}}+e^{-}\to \text{CO}+\text{O}+{\text{e}}^{-}$$

Reaction (11) can be ignored in the present study because of a very low ionization fraction in the plasma, where the estimated value according to measurements by the double probe is approximately 10^−6^. Two other mechanisms definitely contribute to the dissociation phenomenon. According to classical work [19], the mechanism in Reaction (12) is often the most effective channel for CO_2_ dissociation in a non-equilibrium plasma. Namely, the rate coefficient of CO_2_ vibrational excitation by electron impact reaches a maximum value of >10^−8^ cm^3^ s^−1^. At the same time, the loss of vibrational energy by superelastic collisions, or V-T relaxation, is relatively slow [19]. As a result, rather high vibrational temperatures are achieved in CO_2_ plasmas. The cross-section for direct dissociation of the CO_2_ molecule in the ground state Reaction (10) is very low at the electron temperature of a few electron volts so it could probably be ignored. Another mechanism worth mentioning is dissociation of CO_2_ molecules found in metastable electronic states, such as CO_2_ (^1^B_2_) and CO_2_ (^3^B_2_). The densities of such states are unknown in our case, so it is difficult to discuss the contribution of this channel to dissociation of CO_2_ molecules. In any case, there are quite a few possible mechanisms of dissociation by collision with an energetic electron, and the rate of CO and O production in CO_2_ plasma is thought to be similar to the rate of O production in O_2_ plasma. The production mechanisms are therefore not responsible for the rather low dissociation fraction as determined in our experiments for the early afterglow.

Another reason for a rather poor dissociation fraction could be loss rates. An important mechanism for the loss of radicals in the gas phase is recombination by three body collisions. Such a collision is required because the energy and momentum have to be preserved. At around 60 Pa, where the experiments were performed, the probability for three body collisions is low, and gas phase reactions are easily neglected. Radical loss mechanisms that are more important occur on the surfaces. Many authors have shown that the probability for heterogeneous surface recombination of O atoms on glass surfaces is around 10^−4^[12,20]. Less work has been performed on recombination of CO on glass surfaces. Most studies have investigated the average recombination probability for the reactions O + O → O_2_ and CO + O → CO_2_[18,21]. Sepka *et al.* managed to separate the reactions and found out that the dominant surface reaction on a quartz surface in an O_2_-CO mixture was O + O → O_2_[23]. Kolesnikov *et al*. proposed the average catalytic efficiency for both reactions was about 2.5 × 10^−5^ at 300 K for silica-based surfaces [21]. Bykova *et al*. obtained much higher values for quartz surfaces of about 2 × 10^−3^[18]. The difference between the results obtained by Kolesnikov and Bykova is two orders of magnitude. There could be several reasons for this difference, in addition to the simple explanation that the coefficients are not determined precisely. Another, more probable explanation for this difference is the diverse surface properties of the materials investigated by these two groups.

The dissociation fraction is defined as the ratio between the density of a particular radical (e.g., CO) and the density of the stable parent molecules (e.g., CO_2_). The density of the parent molecules is determined from the pressure in the afterglow chamber using Equation (9). The dissociation fraction should therefore decrease with increasing pressure. This has been observed in many afterglow experiments [24], but was not the case in the present study and Figure 7 shows a well-defined maximum on the curve. Some mechanism must prevent a high dissociation fraction at low pressure. This mechanism should exclude any gas phase reactions, because their probability usually increases with increasing pressure. Therefore, the reasons for the rather poor dissociation rate at lower pressure could be explained by surface loss mechanisms. Again, there are several mechanisms for this, including Reactions (1) and (2), but because the coefficients for Reaction (2) are not known, a quantitative calculation could not be performed. However, it is possible to discuss the relatively low dissociation fraction qualitatively in view of the high probabilities for heterogeneous surface oxidation of CO (by Reaction (2)). As mentioned in earlier studies, much higher dissociation fractions were found in detailed characterizations of pure O_2_ afterglows. The anomalous behavior of the CO_2_ afterglow must therefore occur because of the presence of particles other than O or O_2_. In the afterglow of CO_2_ weakly ionized plasma, the only candidate is CO. The recombination coefficient of the CO molecules on borosilicate glass should obviously be higher than the coefficient for recombination of neutral O atoms.

**Figure 8.**
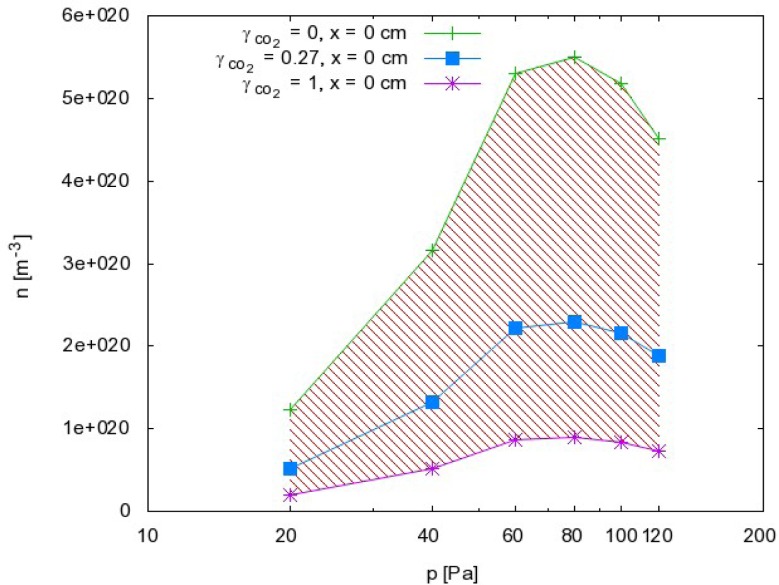
Oxygen atom density versus pressure at 0 cm. The shaded area represents the values that are theoretically possible when taking into account all values of the recombination coefficient. The lowest curve represents the lower limit when the recombination coefficient is 1, and the upper curve represents the upper limit when the recombination coefficient is 0. The curve in the middle represents the O atom density for the most probable value for the recombination coefficient (*i.e*., γ_CO_2__ = 0.27).

From this point of view, we can also discuss the results presented in Figures 5 and 6. Because the probability for heterogeneous surface recombination of CO molecules in the presence of neutral O atoms is unknown, the results presented in Figures 5 and 6 cannot be very accurate. In fact, the results in these figures were obtained by assuming the probability for recombination of CO molecules on the probe tip was the same as the probability for recombination of neutral O atoms Reaction (1). To estimate the error made by this assumption, we have to reconsider Equation (8) and take into account the extreme cases. The recombination coefficient is by definition somewhere between 0 and 1. The O atom density as calculated from Equation (8) obviously depends on the recombination coefficient for Reaction (1) and on the recombination coefficient for Reaction (2). Figure 8 represents the possible range of O atom densities taking into account any value of the recombination coefficient for Reaction (2). The lower limit of the atom density is represented by the highest possible recombination coefficient (*i.e*., γ_CO_2__ = 1). The upper limit is set by taking into account the lowest recombination coefficient (*i.e*., γ_CO_2__= 0). As can be seen from Figure 8, the differences between the lowest and the highest O atom densities were not small. Somewhere in between we can observe the values of the O atom density by taking into account the most probable value of the recombination coefficient (*i.e*., γ_CO_2__= 0.27). Although there is a difference of almost one order of magnitude between the extremes presented in Figure 8, the accuracy of the O atom density determined from the catalytic probe measurements should be good. As stated by Kolesnikov *et al*., the typical recombination coefficient for metals like nickel is between 0.1 and 1. From this point of view, the approximation usually used by all authors, which is that the recombination coefficient for Reaction (2) is equal to the recombination coefficient for Reaction (1), is not only convenient but also justified. The limits of the shaded area in Figure 8 should therefore be taken only as theoretical limits for the worst-case conditions.

The results summarized in Figures 4–6 have practical value because they represent important guidelines for authors working on modification of materials by CO_2_ plasma or afterglow. First of all, Figure 4 clearly shows that material that exhibits catalytic activity for heterogeneous surface recombination for O atoms or CO molecules can be heated to elevated temperatures only by the recombinations. The maximum temperature a sample can reach depends substantially on the position in the afterglow. In very early afterglow, temperatures of about 500 K were detected (Figure 4). Not far from this region (25 cm), the temperature was much lower and this position appears to be better for placing the material if it cannot withstand heating to elevated temperatures. Another possible way to avoid excessive heating of materials is application of lower pressure. Figure 4 clearly shows that heating of a catalytic material is negligible at 20 Pa as long as the sample is placed 5 cm away from the plasma glow region. Here, it is worth noting that such negligible heating should also be obtained at higher pressures. The curves presented in Figure 4 have extremes, and extrapolation to pressures of several 100 Pa should give low temperatures as well. From this point of view, the high pressure extreme would also save catalytic material from overheating. However, it should be stressed that ignition of the radiofrequency electrodeless discharges at elevated pressures is not an easy task.

Materials that do not exhibit catalytic reactivity for surface recombination following Reactions (1) and (2) could be placed anywhere in the afterglow chamber as long as heating is not a concern. As shown in Figures 5 and 6, an appropriate radical density can be selected either by changing the pressure or the position inside the tube. The first few centimeters of the afterglow region (Figure 6) are characterized by large radical gradients, and it is not advisable to place a material in this area. Positions further along the tube are much better for treatment of non-catalytic materials. Figure 6 shows that strong gradients are not present at distances of about 10 cm away from the plasma. Positioning of the sample at around 20 cm would be very suitable for treatment of delicate organic materials where both the flux of radicals on the surface and the thermal load are of concern.

Finally, the right pressure needs to be chosen during treatment of delicate materials by CO_2_ plasma afterglow. Because of uncertainties and for stable treatment, it is always best to operate the apparatus at conditions where a maximum in a certain parameter exists. From this point of view, it is recommended to take into account the results presented in Figure 5. Rather well pronounced maxima are observed on all the curves. If the selected distance from the plasma is 20 cm, the best results in the terms of stability are expected at 100 Pa, which is where the curve in Figure 5 has a maximum. If a higher radical density is chosen, the sample has to be moved closer to the source. In such a case, a different pressure needs to be chosen. Figure 5 indicates that the optimal pressure at 5 cm is 80 Pa, and this would be similar at positions very close to the source. Therefore, there are a variety of experimental conditions that can be chosen for CO_2_ plasma afterglow treatment of materials.

## 4. Conclusions

A catalytic sensor for radical density measurement in CO_2_ plasmas was investigated. For this purpose the dissociation of CO_2_ molecules in a low-pressure weakly ionized plasma, created in a glass tube by an electrodeless radio frequency discharge, was studied using a nickel catalytic probe. Catalytic probes are well known and frequently used sensors for measuring atom densities in discharges of diatomic gas molecules, such as oxygen and hydrogen. With some modification of the recombination model on the probe tip we were able to determine the radical density in an afterglow of an inductively coupled CO_2_ plasma. The nickel catalytic sensor has a very good repeatability and good precision, but a simple recombination model and rough assessment of CO_2_ recombination coefficients on the nickel surface can give rather poor accuracy. For better accuracy a more thorough theoretical model of the CO_2_ heterogeneous recombination must be researched and more accurate CO_2_ recombination coefficients needs to be measured. Nevertheless some useful conclusions can be made from our results. The experimental setup allowed for effective separation of the glowing plasma from the early afterglow. The catalytic probe was mounted in the afterglow region and was movable. This allowed determination of the dissociation fraction along the axis of gas flow through the afterglow tube. The results of systematic measurements showed that the optimal radical density was obtained at a moderate pressure of about 80 Pa, while a well-defined maximum was found for the dissociation fraction at 60 Pa. The observations were discussed in terms of gas phase and surface reactions, and strong radical gradients were qualitatively described by heterogeneous surface loss of gaseous radicals on the walls of the glass chamber. The afterglow of CO_2_ plasma is therefore characterized by a relatively small radical density, and is therefore suitable for treatment of delicate materials.
